# CD4+ T Cell Dependent B Cell Recovery and Function After Autologous Hematopoietic Stem Cell Transplantation

**DOI:** 10.3389/fimmu.2021.736137

**Published:** 2021-09-29

**Authors:** Clarissa Heck, Sophie Steiner, Eva M. Kaebisch, Marco Frentsch, Friedrich Wittenbecher, Carmen Scheibenbogen, Leif G. Hanitsch, Axel Nogai, Philipp le Coutre, Lars Bullinger, Igor-Wolfgang Blau, Il-Kang Na

**Affiliations:** ^1^ Department of Hematology, Oncology and Tumor Immunology, Charité-Universitätsmedizin Berlin, Corporate Member of Freie Universität Berlin, Humboldt-Universität zu Berlin, and Berlin Institute of Health, Berlin, Germany; ^2^ Institute of Medical Immunology, Charité-Universitätsmedizin Berlin, Corporate Member of Freie Universität Berlin, Humboldt-Universität zu Berlin, and Berlin Institute of Health, Berlin, Germany; ^3^ Berlin Institute of Health (BIH), Berlin, Germany; ^4^ Berlin Institute of Health Center for Regenerative Therapies, Charité-Universitätsmedizin Berlin, Berlin, Germany; ^5^ German Cancer Consortium (DKTK), Berlin, Germany; ^6^ Max-Delbrück-Center for Molecular Medicine in the Helmholtz Association, Berlin, Germany; ^7^ Experimental and Clinical Research Center, Berlin, Germany

**Keywords:** B cell defects, autologous hematopoiectic stem cell transplantation, T cell dependent B cell activation, immune reconstitution, multiple myeloma, secondary immunodeficiencies

## Abstract

**Introduction:**

High-dose chemotherapy followed by autologous hematopoietic stem cell transplantation (auto-HSCT) represents a standard treatment regime for multiple myeloma (MM) patients. Common and potentially fatal side effects after auto-HSCT are infections due to a severely compromised immune system with hampered humoral and cellular immunity. This study delineates in depth the quantitative and functional B cell defects and investigates underlying extrinsic or intrinsic drivers.

**Methods:**

Peripheral blood of MM patients undergoing high-dose chemotherapy and auto-HSCT (before high-dose chemotherapy and in early reconstitution after HSCT) was studied. Absolute numbers and distribution of B cell subsets were analyzed *ex vivo* using flow cytometry. Additionally, B cell function was assessed with T cell dependent (TD) and T cell independent (TI) stimulation assays, analyzing proliferation and differentiation of B cells by flow cytometry and numbers of immunoglobulin secreting cells in ELISpots.

**Results:**

Quantitative B cell defects including a shift in the B cell subset distribution occurred after auto-HSCT. Functionally, these patients showed an impaired TD as well as TI B cell immune response. Individual functional responses correlated with quantitative alterations of CD19+, CD4+, memory B cells and marginal zone-like B cells. The TD B cell function could be partially restored upon stimulation with CD40L/IL-21, successfully inducing B cell proliferation and differentiation into plasmablasts and immunoglobulin secreting cells.

**Conclusion:**

Quantitative and functional B cell defects contribute to the compromised immune defense in MM patients undergoing auto-HSCT. Functional recovery upon TD stimulation and correlation with CD4+ T cell numbers, indicate these as extrinsic drivers of the functional B cell defect. Observed correlations of CD4+, CD19+, memory B and MZ-like B cell numbers with the B cell function suggest that these markers should be tested as potential biomarkers in prospective studies.

## Introduction

B cells make up an essential part of the adaptive immune system as drivers of humoral immune responses. Two different pathways leading to B cell activation and thereby the induction of antibody synthesis are often described. These are the T cell dependent (TD *from thymus dependent*) and the T cell independent (TI *from thymus independent*) activation. The TD activation is initiated by protein antigens, which are presented to follicular naïve B cells by antigen presenting cells (APC). Interaction with CD4+ T cells, that previously encountered the same antigen, is necessary in order for naïve B cells to differentiate and synthesize antibodies (1). A germinal center reaction with intense B cell proliferation and repeated B - T cell interaction further induces immunoglobulin class switch and somatic hypermutation to increase antibody affinity (2). During the B - T cell interactions several costimulating factors are essential. For B cell activation CD40L and IL-21 pose to be most relevant (3). TI B cell activation is prompted by mitogens like lipopolysaccharides and bacterial DNA or by polysaccharides on encapsulated bacteria, which activate B cells by crosslinking B cell receptors (BCR). It addresses primarily B cells of the marginal zone (MZ) of the spleen as well as their circulating counterpart (MZ-like B cells *also known as IgM^+^IgD^+^ memory B cells*) and B1 cells (4, 5). The result is a fast and IgM driven B cell response, eliminating pathogens that would otherwise escape a humoral reaction due to the capsule protecting them from phagocytosis by APCs (4).

Autologous hematopoietic stem cell transplantation (auto-HSCT) has been established as a standard treatment regime for patients suffering of multiple myeloma (MM) who are not weakened by major comorbidities (6, 7). After induction therapy, aiming to induce remission of the tumor, stem cell mobilization and apheresis is performed. Shortly before the auto-HSCT, a high-dose chemotherapy with Melphalan is administered in order to eliminate residual tumor cells. This treatment causes a secondary immunodeficiency (SID) in patients by depleting the innate and adaptive immune system. It is leaving patients with a high susceptibility to infections, of which the majority is of bacterial origin (8, 9). Within the first month especially risk factors such as neutropenia and mucosal damage accumulate, being the principal reason for infectious complications and early treatment-related mortality after auto-HSCT (10, 11). After the innate immune system recovered the reconstitution of the humoral and cellular immunity is causing a post-engraftment SID (12). While CD8+ T cell counts recover within one month after auto-HSCT, CD4+ T cells and CD19+ B cells show a delayed recovery (13). Overall B cell numbers recover within 4-8 months after auto-HSCT (14), while certain subpopulations like MZ-like and class switched (CS) memory B cells (memB) take more than a year (15). For the differentiation into CS memB and hence their recovery, CD4+ T cells are essential (16). Quantitatively, CD4+ T cells are known to be the last cell population to recover after auto-HSCT as they require *de novo* synthesis from the thymus and are reduced for more than two years after auto-HSCT (13, 17–19). A more rapid reconstitution of lymphocytes is associated with improved overall survival in MM patients after auto-HSCT (20).

Functionally, hampered serological responses to vaccines have been reported after auto-HSCT in other illnesses. Further, low immunoglobulins and hampered reactions to *in vitro* stimulations especially within the first three months are described (21–24). So far, B cell function could not be recovered *in vitro*. Polyclonal stimulations composed of Staphylococcus aureus Cowan I (SAC), poke weed mitogen (PWM) and oligonucleotides with cytosine and guanin motives (CpG) or CD40L, (IL-21) and CpG that were used in this study have been shown to recover B cell functions in other B cell deficiencies including common variable immunodeficiency (CVID) and systemic autoimmune diseases (25, 26)[Steiner et al. unpublished]. The combination of SAC, PWM and CpG has become an established B cell activation assay introduced by Crotty et al. (27). Similarly, CD40L, IL-21 and CpG described by Cao et al. and Muir et al. have also shown to induce a B cell proliferation and differentiation into immunoglobulin secreting cells (ISCs) and long-lived plasma cells (28, 29). SAC, PWM and CpG measure the TI and TD B cell function in the presence of functional T cells while the combination of CD40L, IL-21 and CpG induces a primarily TD response by substituting costimulatory molecules expressed by CD4+ T cells.

Evoked through the reduced overall survival due to an increased occurrence of infections after auto-HSCT and the significant humoral deficiencies, the aim of this study was to decipher the effect of high-dose chemotherapy and auto-HSCT on the B cell compartment in MM patients in detail. To further distinguish if functional B cell defects are of intrinsic or extrinsic origin, we utilized TD and TI stimulation assays previously discovered to be potent polyclonal B cell activators. This study thereby gives an overview of the phenotypic and functional B cell immunity of patients undergoing treatment for MM especially during early recovery after auto-HSCT. It further lays the foundation for diagnostic and therapeutic strategies targeting SID in patients undergoing auto-HSCT.

## Materials and Methods

### Collection of Peripheral Blood From Healthy Donors and Oncological Patients

For this study peripheral blood was drawn from 11 healthy donors (HD) and 14 MM patients undergoing high-dose chemotherapy and auto-HSCT. Patients’ characteristics are shown in **Supplementary Table 3**. Patients’ peripheral blood was analyzed twice in paired fashion, once right before receiving high-dose chemotherapy (pre-HSCT) and the second assessment was within the first month (range: 14-31 days) after auto-HSCT (post-HSCT). Blood was drawn in heparin tubes and processed within five hours. This study was approved by the ethics committee of Charité - University Medicine Berlin in accordance with the 1964 Declaration of Helsinki and its later amendments (no. EA1/252/14). Patients and HDs gave informed consent.

### Flow Cytometry

Flow cytometry was used in three instances. Firstly, 100µl whole blood was stained for 15 minutes with fluorochrome-conjugated antibodies for a leucocyte panel (**Supplementary Table 1**). Erythrocyte lysis was performed using Erythrocyte lysis buffer (Quiagen, Hilden, Germany) and events/µl detected with the flow cytometer. CD4+ T cell counts were extracted from this data. Second and thirdly, the B cell compartment was characterized by isolation of peripheral blood mononuclear cells (PBMCs) from the remaining whole blood by density gradient centrifugation and analyzed *ex vivo* and after a seven-day stimulation. PBMCs were stained extracellular with fluorochrome-conjugated antibodies (**Supplementary Table 2**) according to a B cell panel adapted from Wehr et al. for CVID patients (30). Dead cells were excluded by staining with DAPI (4′,6-Diamidino-2-phenylindole) (Biolegend, San Diego, USA). All flow cytometric measurements were performed with a Cytoflex S or Cytoflex LX Flow Cytometer (Beckman Coulter, Krefeld, Germany). The data was evaluated using the software Cytexpert version 2.3.0.84 (Beckmann Coulter) and FlowJo version 10.6.2 (BD Biosciences, Franklin Lakes, USA).

### Stimulation of PBMCs

Freshly isolated PBMCs (4x10^6^ cells) were cultured in a 6-well plate. They were diluted in 3ml RPMI medium + 10% fetal calf serum + 1% penicillin/streptomycin (culture medium) and stimulated at 37°C and 5% CO_2_ for seven days. Stimulation protocols were adapted from Crotty et al. and Muir et al. (27, 28). For stimulation two different assays were added. A combination of SAC 1 mg/ml (1:10,000) (Sigma-Aldrich, St. Louis, USA), 100 ng/ml PWM (Sigma-Aldrich, St. Louis, USA), 6 µg/ml CpG - ODN M362 (type C) (Innaxon Biosciences, Tewkesbury, UK) and 50 µM/ml Mercaptoethanol (β-ME) (Sigma-Aldrich) (S/P/C) was used in order to assess TI and TD B cell function in the presence of functional T cells. The second assay comprised 270 ng/ml CD40L (Biolegend), 30 ng/ml IL-21 (ImmunoTools, Friesoythe, Germany) and 6 µg/ml CpG ODN M362 (C/I/C), substituting two costimulatory molecules involved in the TD activation.

### Identification of Immunoglobulin Secreting Cells Using ELISpot Assays

B cell ELISpot assays were performed after seven-day stimulations to quantify immunoglobulin secreting cells (ISCs). 96-well multiscreen filter plates were coated overnight with 1,2 µg/ml goat anti-human IgG (Dianova, Hamburg, Germany), 15 µg/ml goat anti-human IgA (Dianova) or 10 µg/ml goat anti-human IgM (Dianova) primary antibodies in Dulbecco’s phosphate-buffered saline (PBS) (Thermo Fisher Scientific, Waltham, USA). Control wells were coated with PBS only. After blocking of the plates with culture medium and washing, cells were added at concentrations ranging from 1.56x10^3^ over 3.125x10^3^, 6.25x10^3^, 1.25x10^4^, 2.5x10^4^ to 5x10^4^ per 100 µl culture medium per well to identify *Spots* at an optimal dilution. Plates were incubated for four hours, washed and stained with corresponding biotinylated secondary antibodies (IgG and IgM 1:5000 (BD Biosciences), IgA 1:500 (Thermo Fisher Scientific)) overnight. The development of spots was induced by one-hour incubation with 2,5 µg/ml streptavidin-HRP (Biolegend) at room temperature, washing and finally by a combination of 3-amino-9-ethyl-carbazole, Dimethylformamid (1:30) and 3% H_2_O_2_ in acetate buffer (0.3 M sodium acetate solution, 0.2 M acetic acid solution, Aqua dest., pH = 5.0) added for three minutes. The reaction was stopped by rinsing the plates with water. Plates were read and analyzed using the AID ELISpot Reader 7.0.0.0 and the ELISpot software.

### Statistics

Statistical analysis was performed using GraphPad Prism 9.0.1. As data was not normally distributed median and interquartile range (IQR) were calculated for summary statistics. Comparative analysis was performed using nonparametric tests. Patient data to HDs was compared using two-tailed Mann-Whitney-U tests. For dependent samples pre- to post-HSCT and changes upon S/P/C to C/I/C the Wilcoxon test was applied. Groups of three were compared using Kruskal-Wallis-Tests. Correlations were analyzed using the Spearman’s rank correlation test. The correlation coefficient was applied to define weak (0.3<r<0.5), moderate (0.5<r<0.7) and strong (r>0.7) correlations. Exact two-sided statistical significance was defined as a p value <0.05.

## Results

### Treatment-Induced Quantitative B Cell Defects in MM Patients

In our work the B cell compartment of MM patients undergoing high-dose chemotherapy and auto-HSCT was analyzed and compared to HDs. Patients’ characteristics are shown in **Supplementary Table 3**. 64% of patients were male and 36% female and average age was 60 years while in HDs 45% were male and 55% female with an average age of 42 years. The most common MM subtype was IgG MM (64,3%), followed by light chain MM (21,4%) and IgA MM (14,3%). The majority of patients (36%) were diagnosed at stage II, 21% at stage III and 14% at stage I, according to the revised International Staging System (ISS). At the time of high-dose chemotherapy administration 3 patients had achieved complete response, 4 showed a very good partial response, 5 had a partial response and 2 patients only showed a minimal response according to the International Myeloma Working Group consensus (31). HDs were all free from infection within the last 14 days. Pre-HSCT one patient had a reported infection while 10 out of the 14 patients suffered infections within the first month after auto-HSCT, highlighting the increased susceptibility to infections induced through high-dose chemotherapy and auto-HSCT.

**Figure 1:**
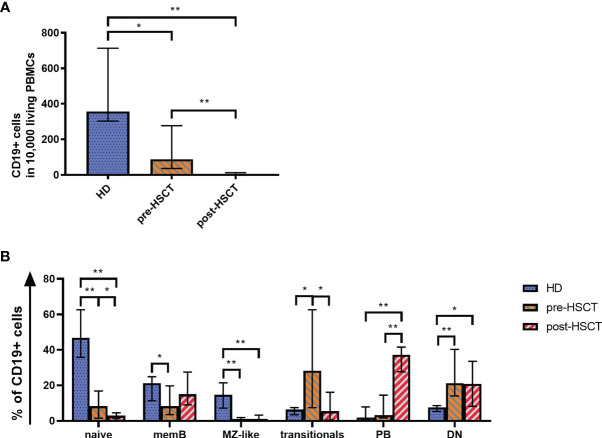
**(A)** Quantitative B cell defects are most pronounced after high-dose chemotherapy and auto-HSCT (post-HSCT). Flow cytometry was used to analyze CD19+ B cell counts per 10,000 PBMCs. **(B)** The reduced B cell compartment shows an altered B cell population distribution ex vivo with a greater proportion of PBs post-HSCT. A comparison of the B cell compartments of HD, pre-HSCT and post-HSCT patients is shown. Using Flow cytometry the percentage of naïve B cells, memB cells, MZ-like cells, double negative (DN) B cells, transitionals and PBs within CD19+ B cells was determined. Bars represent the median ± IQR. Mann-Whitney-U tests were applied to compare HD and patient groups and Wilcoxon tests to compare pre- and post-HSCT with *p < 0.05; **p < 0.001.

Quantitative B cell analysis was performed *ex vivo* by flow cytometry using a B cell panel adapted from Wehr et al. (gating strategy in **Supplementary Figure 1**) (30). Measurements showed significantly reduced CD19+ cell counts in patients before and most pronounced after HSCT compared to HD (pre-HSCT: p=0.007; post-HSCT: p<0.001) (**Figure 1A**). Pre-HSCT patients had previously undergone induction therapy in most cases consisting of Bortezomib, Cyclophosphamide and Dexamethasone (VCD) as well as stem cell mobilization with Cyclophosphamide and Granulocyte colony-stimulating factor (G-CSF) administration. A detailed overview of patients’ treatment background can be found in **Supplementary Table 3**. In these patients the median count of CD19+ cells was at 88.5 [36.0-277.3] CD19+/10,000 PBMCs (median [IQR]), which corresponds to a quarter of counts detected in HD (HD: median=357 [303-713] CD19+ cells/10,000 PBMCs). However, reduced numbers of CD19+ cells (212 [154.5-260] CD19+ cells/10,000 PBMCs) were also found in untreated MM patients highlighting quantitative B cell defects prior to any treatment (data not shown) (19). The high-dose chemotherapy with Melphalan followed by auto-HSCT entailed an almost complete depletion of CD19+ B cells, resulting in a median drop of 95% from pre-HSCT to 4.1 [1.6-12.25] CD19+ cells/10,000 PBMCs post-HSCT (p<0.001).

### Treatment-Induced Alterations in the Distribution of B Cell Subpopulations

Consistent with the total CD19+ B cells, all measured B cell subpopulations decreased significantly from pre-HSCT to post-HSCT (**Supplementary Figure 2**). However, the treatments affected B cell subpopulations to different degrees, which led to altered B cell subset distributions as it can be seen in **Figure 1B**. The distribution of subpopulations in the HD group encompassed 56% of total naïve cells out of which 6% comprised transitional B cells. IgM only and CS memB together made up 21%, while MZ-like B cells covered almost 15%. Double negative B cells (DN) reached a median of 8%. Plasmablasts (PBs), which represent the smallest population in healthy individuals, made up <2%. An altered subset distribution became apparent in MM patients who had undergone induction treatment. A disproportional and significant reduction was observed of especially MZ-like B cells (p<0.001), which subsequently made up only 1% of B cells. Further, the share of mature naïve and memB cells was down to 8% of CD19+ cells each (naïve p<0.001, memB p=0.037). PBs and most markedly transitionals gained a disproportionally high percentage, with transitionals rising up to around 1/3 of the B cells (p=0.013). Following high-dose Melphalan and auto-HSCT the most eminent change to pre-HSCT was seen in a rise of PBs to 37% of the B cells (p<0.001). In contrast to a healthy distribution, the compartment post-HSCT comprised a higher proportion of memB cells (15%) than naïve B cells, which sank to 3% (pre- to post-HSCT p=0.041). In this early reconstitution phase transitionals remained at 5%. DN were disproportionally high pre- and post-HSCT at a median of 21% (pre-HSCT p<0.001, post-HSCT p=0.21).

### *Ex Vivo* Stimulation With CD40L/IL-21 Partially Restores B Cell Proliferation

**Figure 2:**
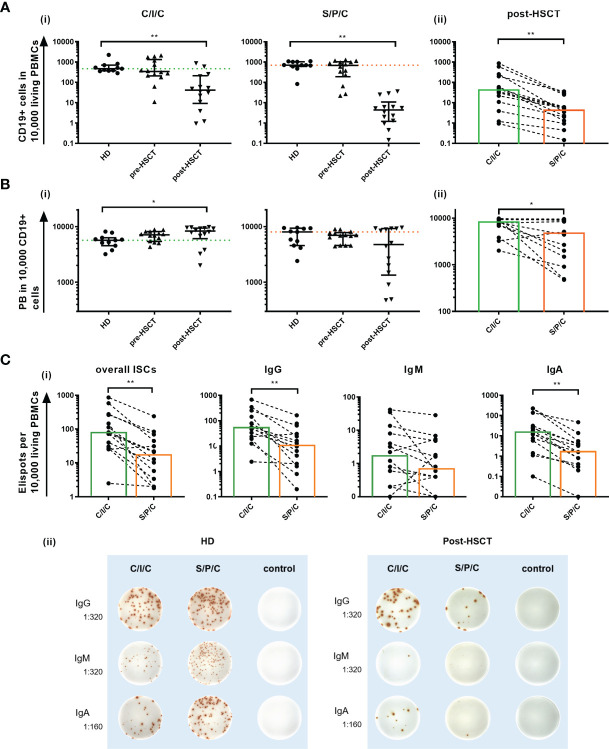
**(A)** *Ex vivo* C/I/C stimulation can partially reverse the quantitative B cell defect. **(i)** Comparison of CD19+ counts/10,000 PBMCs between HD, pre-HSCT and post-HSCT patients measured in flow cytometry after either C/I/C or S/P/C stimulation. Mann-Whitney-U tests were performed. Bars represent median ± IQR. The dotted lines mark the median of HDs upon C/I/C (green) or S/P/C (orange). **(ii)** Post-HSCT measurements of CD19+ cells/10,000 PBMCs upon C/I/C or S/P/C stimulation were compared with the Wilcoxon test. Boxes mark the median counts after C/I/C (green) or S/P/C stimulation (orange). All graphs in **(A–C)** are plotted on a log 10 scale and *p < 0.05, **p < 0.001. **(B)** Differentiation to PBs is hampered after S/P/C stimulation post-HSCT, but appears functional after C/I/C stimulation. **(i)** The differentiation into PBs is evaluated by analyzing PBs per 10,000/CD19+ cells measured by flow cytometry upon C/I/C or S/P/C stimulation. Mann-Whitney-U tests were performed for comparing HD and patients. The dotted lines mark the median of HDs upon C/I/C (green) or S/P/C (orange). Bars represent the median ± IQR. **(ii)** Wilcoxon test is applied to compare post-HSCT PBs per 10,000/CD19+ cells between stimulation assays. Boxes mark the median counts after C/I/C (green) or S/P/C stimulation (orange). **(C)** Differentiation to ISCs is hampered after S/P/C stimulation post-HSCT, but appears regular after C/I/C stimulation. **(i)** Differentiation into ISCs was assessed by identifying ELISpots per 10,000 added cells, assuming one spot equals one ISC. Here the amount of spots generated upon C/I/C or S/P/C stimulation was compared using the Wilcoxon-test in post-HSCT patients’ samples. Boxes mark the median counts after C/I/C (green) or S/P/C stimulation (orange). **(ii)** Representative ELISpot wells of a HD and a post-HSCT patient upon C/I/C or S/P/C stimulation and control wells. Dilutions refer to the amount of PBMCs that were added per well.

With the intention to decipher functional B cell defects in MM patients undergoing auto-HSCT and to further distinguish between B cell intrinsic and extrinsic causes for the hampered function, *ex vivo* B cell stimulation assays were performed. As a common marker for successful B cell activation, B cell proliferation was assessed by comparing CD19+ counts measured in flow cytometry before and after the stimulations. Patients**’** samples exhibited a higher proliferation than HDs with the strongest CD19+ increase upon C/I/C stimulation post-HSCT (x9.56). An exception was a reduction to the S/P/C stimulus post-HSCT (x0.86) (**Table 1**). To evaluate whether these induction rates served to recover B cell numbers, CD19+ cells/10,000 PBMCs after stimulation were compared between HD and the patients (**Figure 2Ai**). Pre-HSCT, both the C/I/C as well as the S/P/C stimulation induced a proliferation strong enough to compensate the quantitative deficit *ex vivo*, reaching a median of CD19+ cells that did not differ significantly from HD (C/I/C: median HD=471 [374-704], median pre-HSCT=343 [207.8-1325]; S/P/C: median HD=706 [610-1030], median pre-HSCT=687[191-1032]). Post-HSCT the initial deficit was too extensive for either stimulation to compensate, causing the median CD19+ counts to remain significantly reduced in comparison to HDs (C/I/C and S/P/C p<0.001). However, there was a great difference between the two stimulation assays. C/I/C provoked a strong proliferation to a median of 41.5 [9.2-212.5] CD19+/10,000 PBMCs and led to normalized CD19+ counts in 4 patient samples. The S/P/C stimulation in contrast did not provoke any proliferation and remained on a median of 4.4 [1.2-10.9] CD19+/10,000 PBMCs. Overall C/I/C showed a 10 times higher CD19+ count post-HSCT than S/P/C (p<0.001) (**Figure 2Aii**) and could partially reverse the CD19+ deficit.

**Table 1:** Median induction rates of B cell populations upon C/I/C or S/P/C stimulation.

	HD		Pre-HSCT		Post-HSCT	
	C/I/C	S/P/C	C/I/C	S/P/C	C/I/C	S/P/C
**CD19+**	1,18	1,51	5,51	4,56	9,56	0,86
**PB**	28,47	42,93	21,57	16,50	2,10	1,77
**IgM PB**	16,11	22,89	7,59	87,08	1,25	0,87
**CS PB**	15,59	14,32	12,23	3,55	1,61	0,86
**memB**	0,24	0,03	0,27	0,08	0,07	0,35
**IgM memB**	0,53	0,04	0,24	0,23	0,05	0,07
**CS memB**	0,21	0,04	0,21	0,06	0,07	0,39
**naive**	0,23	0,12	0,38	0,14	0,34	0,31
**transitionals**	0,04	0,02	0,01	0,01	0,23	0,03
**MZ-like**	0,16	0,01	1,44	0,36	0,22	1,96

Green fields indicate a positive induction; red fields a reduction of the cell population. Shown are CD19+ cells per 10,000 PBMCs and B cell subpopulations per 10,000 CD19+ cells.

### *Ex Vivo* Stimulation With CD40L/IL-21 Partially Restores Differentiation Into PBs and ISCs

A second parameter to assess B cell function was differentiation into PBs and ISCs upon stimulation.

To measure differentiation into PBs the change of distribution of B cell subpopulations within the CD19+ compartment upon stimulation was determined. In HDs a successful B cell activation was marked by a reduction of memB, naïve, transitional and MZ-like cells through apoptosis and differentiation into PBs, which consequently increased (**Table 1**) (32–34). The TD focused C/I/C assay led to a stronger induction of CS PB and the combined TI activating assay S/P/C induced a bigger increase of IgM PBs in HDs and pre-HSCT however, failed to do so post-HSCT (**Table 1**).

The overall count of PB/10,000 CD19+ cells was compared between HDs and patients to determine a successful differentiation into PBs (**Figure 2B**). Since this parameter is reflecting the B cell function for existing B cells only, it remains unaffected by the quantitative defect. In this setting, the C/I/C stimulation generates PBs to healthy levels before and after auto-HSCT with post-HSCT levels even significantly exceeding those of HDs (p=0.02). Since C/I/C activates especially memB cells, this is most likely due to a higher memB cell proportion post-HSCT forming a more extensive response (35). The S/P/C stimulation on the other hand resulted in a weaker PB generation especially post-HSCT (HD: median=8020 [4560-9330]; post-HSCT: median=4760 [1345-9530]; p=0.23 ns) with high variability among the patients (IQR=1345-9530). Post-HSCT PB counts upon C/I/C significantly exceeded those upon S/P/C (C/I/C: median=8310 [6068-9423]; S/P/C: median=4760 [1345-9530]; p=0.01) bringing to light a hampered B cell function upon S/P/C whereas differentiation into PBs is fully restored upon C/I/C.

Differentiation into ISCs, measured in B cell ELISpots, confirmed an IgM weighted response upon S/P/C in HD and pre-HSCT and IgG and IgA weighted upon C/I/C in all groups with no significant differences in the overall ISCs (data not shown). Post-HSCT ISCs were reduced compared to HD, which could be attributed to the lesser amount of B cells. However, a deficient differentiation upon S/P/C was confirmed with significantly less overall ISCs compared to C/I/C (S/P/C: median=17 [3-51]; C/I/C: median=76 [29-266]; p<0.001), less IgG (S/P/C: median=11 [2-39]; C/I/C: median=56 [21-198]; p<0.001) and less IgA (S/P/C: median=2 [0-5]; C/I/C: median=15 [5-58]; p<0.001) ISCs upon S/P/C than C/I/C. In fact even IgM ISCs were reduced upon S/P/C (S/P/C: median=1 [0-5]; C/I/C: median=2 [0-9]; p=0.05) indicating a strongly deficient immunoglobulin production upon S/P/C stimulation (**Figure 2C**).

### B Cell Proliferation and Differentiation Upon Stimulation Correlate With *Ex Vivo* CD4+, CD19+, memB and MZ-Like Counts

Initiated through the variance in B cell responses to the stimulations in post-HSCT patients, correlations between the counts of relevant B cell populations *ex vivo* and the measured parameters for B cell function were calculated (**Table 2**). Both stimulations showed a moderate to strong correlation between the B cell function and the total number of CD19+ B cells. MemB cells showed a stronger correlation with the PB induction upon C/I/C than S/P/C stimulation and only a weak influence on the ISC production in both stimulations. Overall, the total numbers of CD19+ cells as well as memB cells were identified as correlating parameters to whether B cell activation was successful. MZ-like B cells served as an additional parameter to evaluate the response to S/P/C and hence could predict successful TI response. A moderate to strong correlation was measured between the differentiation of B cells upon S/P/C and the count of MZ-like B cells. The influence on proliferation was low. Upon C/I/C, MZ-like cells only showed a moderate correlation with PB/CD19+ cells hinting to a low relevance of the MZ-like cells and hence the TI response upon this assay.

**Table 2:** Correlation of cell counts *ex vivo* and parameters for B cell functionality.

		C/I/C		S/P/C	
cell population *ex vivo*	functional parameter	p-value	correlationcoefficient (r)	p-value	correlation coefficient (r)
**CD19+**	CD19+/PBMC	0,005*	0,724	<0,001*	0,887
	PB/CD19+	0,001*	0,790	0,046*	0,546
	PB/PBMC	0,002*	0,770	0,001*	0,788
	overall ISCs	0,009*	0,682	0,039*	0,562
**memB**	CD19+/PBMC	0,027*	0,596	0,006*	0,710
	PB/CD19+	0,006*	0,706	0,192	0,371
	PB/PBMC	0,015*	0,644	0,035*	0,574
	overall ISCs	0,087	0,477	0,096	0,464
**MZ-like**	CD19+/PBMC	0,381	0,252	0,164	0,394
	PB/CD19+	0,055	0,528	0,004*	0,744
	PB/PBMC	0,237	0,336	0,008*	0,693
	overall ISCs	0,177	0,382	0,007*	0,701
**CD4+**	CD19+/PBMC	0,016*	0,692	0,07	0,546
	PB/CD19+	0,020*	0,671	0,528	0,203
	PB/PBMC	0,012*	0,713	0,379	0,280
	overall ISCs	0,016*	0,692	0,342	0,301

faint green: weak correlation; medium green: moderate correlation; dark green: strong correlation. The correlation coefficient (r) was used to divide relationships into weak (0.3<r<0.5), moderate (0.5<r<0.7) and strong (r>0.7) correlations. *p < 0.05.

### CD40L/IL-21 Restore CD4+ T Cell Dependent B Cell Function

Due to the significant differences in B cell responses between the C/I/C and S/P/C assay post-HSCT, the divergence between the two assays was further investigated. Since CD40L and IL-21 are costimulatory molecules usually expressed on CD4+ T cells *in vivo* a dependence of the B cell recovery and differentiation on CD4+ T cells was assessed. Hence, additionally the correlation between the B cell function and CD4+ cells was calculated. Upon the C/I/C stimulation, all functional parameters showed a moderate to strong correlation to the count of CD4+ T cells while they only had some relevance for the proliferative outcome upon S/P/C (**Table 2**). Since CD4+ T cells are essential players in the TD B cell activation a correlation with CD4+ T cell counts suggested a recovered TD activation under C/I/C.

**Figure 3:**
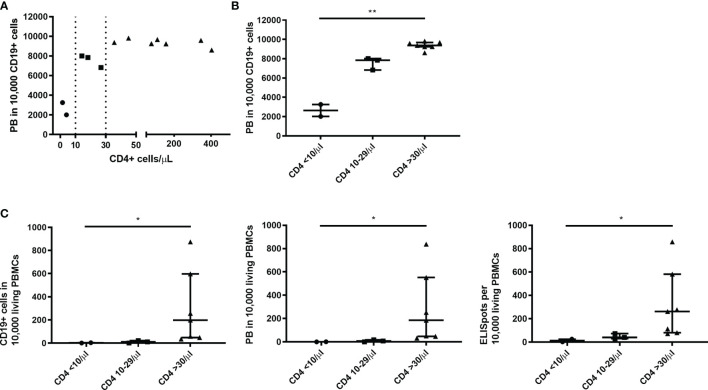
Thresholds of 10 and 30 CD4+ T cells/µl were associated with an improved B cell function upon C/I/C stimulation. **(A)** A spearman correlation between the count of CD4+ T cells (cells/µl) *ex vivo* and the functional parameter PB/10,000 CD19+ cells revealed two thresholds of 10 cells/µl and 30 cells/µl, marked here with dotted lines. **(B)** Thresholds are highlighted by splitting samples into three groups according to the CD4+ count: CD4 <10/µl; CD4 10-29/µl; CD4 ≥30/µl plotted against PB/10,000 CD19+ cells. **(C)** Three groups of CD4+ counts plotted against other functional parameters: CD19+/10,000 PBMCs; PB/10,000 PBMCs; ISCs/10,000 PBMCs. **(B, C)** Bars represent median ± IQR. Groups were compared by Kruskal-Wallis-Test with *p < 0.05 and **p < 0.001.

Looking at the PB counts per CD19+ cells - the most sensitive marker for B cell function in this study - two thresholds of CD4+ cells could be identified to be associated with a better B cell function upon C/I/C stimulation (**Figure 3A**). These were at 10 cells/µl and 30 cells/µl, splitting the patients in three groups: 1) CD4+ cells <10/µl (n=2), 2) 10-29 cells/µl (n=3) and 3) ≥30 cells/µl (n=7). In combination with the C/I/C stimulation CD4+ cell counts between 10 and 29 cells/µl sufficed to mount an effective PB response, however not as successful as CD4+ cell counts above 30 cells/µl. Comparing these three groups, there was a significant increase of PB/10,000 CD19+ cells (p<0.001) (**Figure 3B**). Since PBs were measured per 10,000 cells, numbers could not increase above 10,000, possibly covering up a continuous increase. The relevance of the two thresholds could also be seen when comparing the groups to other functional parameters analyzed in this work (CD19+/PBMCs: p=0.002; PB/PBMCs: p=0.001; ISCs/PBMCs: p=0.004) (**Figure 3C**).

## Discussion

Lymphocyte reconstitution following auto-HSCT is an essential factor to protect MM patients from infections and tumor relapse and can hence contribute to reduce morbidity and mortality of these patients (36). This study further deciphered a specific pathomechanism underlying the slow B cell recovery after auto-HSCT and additionally detected alterations that could pave the way for new diagnostic and therapeutic strategies.

SID after auto-HSCT reportedly does not differ significantly between MM patients and other underlying diseases. This has been investigated in regard to infections (37) and could be concluded by comparing our work and a study from Gernert et al. in regard to the composition of the B cell compartment (15). This suggests that results shown in this work could be transferred to patients with various illnesses who are undergoing treatment with auto-HSCT. However, the limited patient cohort size underscores the exploratory nature of the study.

Quantitative B cell defects contributing to the increased susceptibility to infections in treated MM patients have previously been described and are most prominent after auto-HSCT (19). The present study measured almost complete depletion of peripheral blood B cells following high-dose chemotherapy and auto-HSCT. This has also been described in other diseases and is attributed to the cytoreductive high-dose chemotherapy with Melphalan that is especially affecting immune cells (19, 38). The partial recovery of B cell counts through proliferation upon *ex vivo* C/I/C stimulation in this study suggests an additional functional (qualitative) defect contributing to the quantitative deficit. Because *ex vivo* stimulation was able to trigger efficient proliferation of patients’ B cells, an extrinsic rather than intrinsic cause seems likely.

All measured B cell subpopulations were significantly reduced post-HSCT. A shift in the distribution of subpopulations within the B cell compartment contributed further to the B cell deficiency. The major proportion of the B cell compartment post-HSCT consisted of PBs and DN B cells while populations that can respond to new pathogens such as naïve, MZ-like and transitional B cells comprised a smaller share. Gernert et al. observed a similar composition of the B cell compartment after the first month following auto-HSCT in patients with systemic sclerosis (15). Gernert et al. noted a higher percentage of transitional B cells though, most likely due to the fact that in our study blood from most patients was obtained earlier than one month after auto-HSCT at which point reconstitution was not as progressed (39). The high proportion of PBs most likely reflects the acute systemic inflammation reaction following the high-dose chemotherapy, weakening the immune system in addition to the quantitative defects (40).

In order to further address intrinsic versus extrinsic factors for B cell defects after auto-HSCT, polyclonal stimulation assays were used to analyze the TD and TI B cell function. While the results deliver information about general B cell function a limitation of this study is, that they do not necessarily reflect antigen specific responses. The functional analysis revealed a reduced B cell function within the first month post-HSCT, which was marked by a hampered immune response upon S/P/C. This assay served as a control since it induces TI and TD B cell activation in the presence of functional T cells. SAC induces a TI activation enhanced by CPG, which can be seen in a response with IgM PBs and ISCs (27, 41). PWM delivers additional signals for a TD stimulation inducing class switch and differentiation, reflected primarily in CS PBs and ISCs (42, 43). A significantly hampered differentiation into PBs and ISCs of all immunoglobulin subclasses led to the conclusion of a defect TD and TI response. The mitogens SAC and PWM have previously been tested after auto-HSCT and bone marrow transplantation in leukemia patients. These studies also observed a hampered proliferation upon stimulation within the first three months and in certain cases beyond that (21, 22, 44). Based on the fact that B cell function could not be recovered *in vitro* an intrinsic B cell defect was previously assumed (21, 23).

In contrast, CD19+ recovery and differentiation into primarily CS PBs and ISCs upon C/I/C stimulation implied a TD response recovery upon extrinsic factors. Correlations of functional outcomes with CD4+ cells confirmed a stronger TD response with higher CD4+ T cell counts. This highlighted the effective T cell help provided by the present CD4+ cells upon C/I/C stimulation. Two thresholds at 10 cells/µl and 30 cells/µl could be identified suggesting low levels of CD4+ cells suffice to provide effective help in this setting. However, these results should be interpreted with caution until confirmed in a larger cohort, since the sample size particularly for the group <10 CD4+ cells/µl was very limited.

CD4+ T cells are known to be reduced in MM patients after undergoing auto-HSCT (13, 19) and have been described to contribute to a hampered TD B cell response. However, CD4+ cell counts did not correlate with a better function upon S/P/C stimulation, which contains the TD dependent stimulant PWM. This is highlighting the restorative effect that the stimulants CD40L and IL-21 have on the TD B cell function. CD40L and IL-21 are costimulatory molecules primarily expressed by CD4+ T cells *in vivo* and are indispensable in the T - B cell interaction (45, 46). The restorative effect on the TD B cell function of these molecules implies a distorted T - B interaction in patients recovering from high-dose therapy and auto-HSCT. This is further supported by similarities of their SID concerning B cell function and clinical presentation to other immunodeficiencies. Patients with CVID or ICOS deficiency also show a reduced ISC generation upon stimulation (25) and suffer of hypogammaglobulinemia, a reduced germinal center reaction and a reduced serological response due to an inefficient T cell help to B cells (47).

The costimulatory factor IL-21 is also known to be reduced after auto-HSCT (48). Details about the expression of CD40L on activated T cells early after HSCT are missing. However, it has been described that the expression is reduced on cord blood T cells (49) and that reconstitution after HSCT follows the ontogenetic development (50). A reduced CD40L expression and IL-21 secretion could hence influence the T - B interaction post-HSCT. *In vitro* CD40L enhances intercellular adhesion and both CD40L and IL-21 have shown to induce B cell proliferation as well as differentiation into ISCs including immunoglobulin class switch (41, 51–53). The stimulation with CD40L and IL-21 in our study could have provoked a synergistic function of the existing CD4+ T cells and the stimulants, restoring the T - B interaction and explaining the successful TD response upon the C/I/C in contrast to the S/P/C assay.

The mechanism by which CD40L and IL-21 restore TD B cell activation could be based on affecting and altering suppressive mechanisms. A suppressive effect of T cells on antibody production has been described after HSCT (44). A recent study on B cell function in systemic autoimmune diseases revealed a B cell hyporesponsiveness through chronic *in vivo* stimulation without T cell help through CD40-CD40L interaction. This resulted in decreased phosphorylation of BCR-related signaling molecules. CD40L stimulation *in vitro* increased BCR signaling and induced proliferation in contrast to a hampered response to CpG only (26). Whether a similar pathomechanism could be involved after auto-HSCT and whether stimulation with CD40L and IL-21 affects phosphorylation patterns in patients after auto-HSCT should be explored in prospective studies.

Whether the restorative effect is confined to TD B cell function or if also TI function can be improved through CD40L and IL-21 needs to be further investigated. A great variance especially in the PB/CD19+ B cell response upon S/P/C correlated with the number of MZ-like cells, which are known to be key players in the TI B cell response (4, 5) and have been described to be significantly and long-term reduced after auto-HSCT (15). Although acting T cell independent, specific interactions such as CD40-CD40L in the presence of IL-21 have also been discussed for inducing CS of immunoglobulins produced by MZ-like cells (32). However, if the TI response post-HSCT was only lacking CD40L-CD40 interaction, an IgM response upon S/P/C stimulation would have been expected.

Based on the high variability of B cell responses among patients within and in between the TD and TI assays certain cell populations were investigated as predictors for the B cell response *ex vivo*. Both stimulation assays showed a correlation of CD19+ and memB cells with the overall B cell function. While the primarily TD activating C/I/C assay correlated with the number of CD4+ T cells, the B cell function upon the also TI activating S/P/C assay correlated with the MZ-like cells. A previous work observed a correlation between CD4+ and CD19+ cell counts with opportunistic infections after auto-HSCT (19). In young children, splenectomized patients and CVID patients, the reduction of MZ-like cells goes along with a higher susceptibility to TI antigens such as encapsulated bacteria like streptococcus pneumoniae or haemophilus influenza, which also affect patients after HSCT (54–56). These findings suggest CD19+ and memB cells as parameters for determining general B cell function early after HSCT and CD4+ cells for TD and MZ-like cells for TI B cell function. Assessment of these cell populations should therefore be further evaluated to whether they potentially serve as diagnostic parameters for predicting B cell function in patients early after auto-HSCT. The detected CD4+ cell thresholds moreover suggest that diagnostic levels could be identified *in vivo*.

## Conclusion

Quantitative and functional B cell defects occur after high-dose chemotherapy and auto-HSCT. Efficient B cell proliferation and differentiation upon TD *ex vivo* stimulation highlight the role of extrinsic roots for the functional defect. Correlations of the TD B cell function with CD4+ T cell counts highlight the restorative effect of the applied stimulants CD40L and IL-21. The *in vivo* role of CD40L and IL-21 suggests a hampered B - T interaction as the underlying defect. By further deciphering the pathomechanism involved in B cell defects after auto-HSCT, these findings contribute new elements to the constant search of ways to improve immune reconstitution after auto-HSCT. Besides CD4+ T cells, also numbers of B cells, memB cells and MZ-like B cells correlated with B cell function *ex vivo*. Quantitative assessment of these cell populations should further be explored as potential biomarkers for estimating B cell function in patients early after auto-HSCT.

## Data Availability Statement

The raw data supporting the conclusions of this article will be made available by the authors, without undue reservation.

## Ethics Statement

The studies involving human participants were reviewed and approved by Charité’s Ethics Committee. The patients/participants provided their written informed consent to participate in this study.

## Author Contributions

CH participated in the research design and patient acquisition, performed experiments and data analysis and wrote the article. I-KN substantially contributed to the research design, patient acquisition, data analysis and the writing of the article. SS and MF were involved in the research design and data analysis. FW participated in the recruitment of patients. CS, LH, and EK contributed to the research design. I-WB and LB discussed and finalized the data analysis. AN and PC provided patient samples. All authors contributed to the article and approved the submitted version.

## Funding

The project was financed through grants recieved by I-KN from Takeda Pharmaceutical Company I Shire (grant ID number IIR-DEU-1589), Octapharma (grant number: SAP-Nr. 125051) and the Berlin Institute of Health Johanna Quandt Professorship. The funders were not involved in the study design, collection, analysis, interpretation of data, the writing of this article or the decision to submit it for publication. The library of Charité University Medicine Berlin supports the open access publication.

## Conflict of Interest

I-KN declares a conflict of interest through financial support from Shire/Takeda and Octapharma.

The remaining authors declare that the research was conducted in the absence of any commercial or financial relationships that could be construed as a potential conflict of interest.

## Publisher’s Note

All claims expressed in this article are solely those of the authors and do not necessarily represent those of their affiliated organizations, or those of the publisher, the editors and the reviewers. Any product that may be evaluated in this article, or claim that may be made by its manufacturer, is not guaranteed or endorsed by the publisher.
