# Supplementary material for: CD4+ T Cell Dependent B Cell Recovery and Function After Autologous Hematopoietic Stem Cell Transplantation
Source: Front Immunol. 2021 Sep 29;12:736137. doi: 10.3389/fimmu.2021.736137 (PMC8519398; doi:10.3389/fimmu.2021.736137)
Supplement: Supplementary file 1 [file DataSheet_1.docx]

Supplementary Material

# Supplementary Data

## Supplementary Tables

Table 1: Flow cytometry antibodies used for leucocyte panel

| Target epitope | Fluorochrome-conjugate | Dilution x:100 |
| --- | --- | --- |
| CD25 | APC | 2 |
| CD3 | A700 | 0,5 |
| CD16 | APC-750 | 2 |
| CD127 | PE | 1 |
| CD56 | PE Dazzle | 0,5 |
| CD123 | PE-Cy7 | 0,5 |
| IgD | FITC | 0,5 |
| HLA-DR | PerCP-C5,5 | 1 |
| CD27 | BV421 | 0,5 |
| CD4 | BV510 | 2 |
| CD19 | BV605 | 0,5 |
| CD14 | BV650 | 0,5 |
| CD8 | BV785 | 0,5 |
| CD45 | BUV395 | 1 |

Table 2: Flow cytometry antibodies used for B cell panel

| Target epitope | Fluorochrome-conjugate | Dilution x:100 |
| --- | --- | --- |
| CD19 | PE-Cy7 | 0,5 |
| IgD | APC-H7 | 0,5 |
| CD3 | PB | 1 |
| IgG | PE | 1 |
| CD27 | FITC | 1,5 |
| CD38 | AF700 | 1,5 |
| IgM | APC | 2 |
| CD24 | PerCP-C5,5 | 2 |
| Live/dead | Dapi | 1 |

Table 3 Clinical backgrounds of patients undergoing auto-HSCT

| Patient ID | Gender (m/f) | Age in years | MM subtype | Treatment preceding high-dose chemotherapy and auto-HSCT | Infections requiring treatment | | Remis-sion status* | Days after HSCT |
| --- | --- | --- | --- | --- | --- | --- | --- | --- |
| P001 | m | 71 | IgG Kappa | Dexamethasone pulse therapy 7x plasmapheresis 2x MTX, Dexamethasone i.t. 4x VCD 3x Rituximab 3x Lenalidomide, Dexamethasone Cyclomob. + G-CSF | Pre-HSCT: -Aspergillus pneumonia  Post-HSCT: -C. diff. Colitis -E. coli sepsis | PR | | 20 |
| P002 | m | 60 | IgG Kappa | Radiatio of cervical vertebrae 4x VCD Cyclomob. + G-CSF | Post-HSCT:  -Staph. epidermidis catheter sepsis | MR | | 18 |
| P003 | m | 47 | IgG Lambda | 4x VCD Cyclomob. + G-CSF 1. Melphalan +HSCT | Post-HSCT:  -FUO during neutropenia | VGPR | | 14 |
| P004 | m | 53 | Lightchain Lambda | 4x Bortezomib/Revlimide/ Dexamethasone Cyclomob. + G-CSF | Post-HSCT: -Staph. epidermidis sepsis | CR | | 17 |
| P005 | m | 69 | Lightchain Lambda | 3x VCD Cyclomob. + G-CSF | none | VGPR | | 20 |
| P006 | f | 69 | IgG Lambda | 4x AD Cyclomob. 1. Melphalan +HSCT 1x VCD 2. Melphalan +HSCT  2x VCD Cyclomob. + G-CSF | Post-HSCT:  -herpes genitalis | CR | | 17 |
| P007 | f | 54 | IgG Kappa | 6x IKRD (Anti-CD38, Carfilzomib, Lenalidomide, Dexamethasone) Cyclomob. + G-CSF | none | CR | | 21 |
| P008 | m | 64 | IgG Kappa | Radiatio of lumbar vertebrae 4x VCD Cyclomob. + G-CSF | Post-HSCT:  -RSV-pneumonia | PR | | 22 |
| P009 | f | 55 | IgG Kappa | 4x VCD Cyclomob. + G-CSF | none | MR | | 21 |
| P010 | m | 67 | IgG Kappa | Radiatio of hip 6x VCD Cyclomob. + G-CSF | Post-HSCT: -catheter associated abscess | PR | | 17 |
| P011 | f | 56 | IgA Kappa | 4x VCD Cyclomob. + G-CSF 1. Melphalan +HSCT | none | PR | | 14 |
| P012 | f | 68 | IgA Lambda | 3x VCD Cyclomob. + G-CSF | Post-HSCT:  -FUO during neutropenia | PR | | 19 |
| P013 | m | 59 | IgG Lambda | 3x VCD Cyclomob. + G-CSF | Post-HSCT:  -FUO during neutropenia -C. diff. colitis | VGPR | | 29 |
| P014 | m | 48 | Lightchain Kappa | 4x VCD Cyclomob. + G-CSF | Post-HSCT:  -Influenca-pneumonia -sepsis | VGPR | | 31 |

Abbreviations: m: male; f: female; MTX: methotrexate; i.t.: intrathekal; Cyclomob.: Cyclophosphamide mobilisation; AD: Adriamycin, Dexamethasone; C. diff.: Clostridium difficile; E. coli: Escherichia coli; Staph.: staphylococcus; FUO: fever of unknown origin; RSV: respiratory syncytial virus; CR: complete response; VGPR: verg good partial response; PR: partial response; MR: minimal response
* remission status at sampling pre-HSCT according to the International Myeloma Working Group consensus

## Supplementary Figures

##

Figure 1 Gating strategy for B cell phenotyping in flow cytometry. Example of the gating performed in a HD.

**Figure 2 Quantitative reductions of all B cell subpopulations post-HSCT.** Comparison of B cell subpopulation counts per 10,000 PBMCs between HD, pre-HSCT and post-HSCT patients. Bars represent median ± IQR. Mann-Whitney-U test was performed for comparison of HDs and patients and Wilcoxon test was applied for comparison among patients with *p<0.05, **p<0.001.
